# Patterned few nanometer-thick silver films with high optical transparency and high electrical conductivity†

**DOI:** 10.1039/d1ra00549a

**Published:** 2021-03-19

**Authors:** Xie He, Qijie Cao, Jing Pan, Liu Yang, Sailing He

**Affiliations:** Centre for Optical and Electromagnetic Research, National Engineering Research Center for Optical Instruments, Zhejiang University Hangzhou 310058 China optyang@zju.edu.cn sailing@zju.edu.cn; Ningbo Research Institute, Zhejiang University Ningbo 315100 China; JORCEP, School of Electrical Engineering, Royal Institute of Technology (KTH) S-100 44 Stockholm Sweden

## Abstract

Transparent conductive electrodes (TCEs) are experimentally demonstrated using patterned few nanometer-thick silver films on zinc oxide-coated rigid and flexible substrates. The grid lines are completely continuous, but only 8.4 nm thick. This is the thinnest metallic grid we are aware of. Owing to the high transparency of both the grid lines and spacing, our TCE with an opening ratio (OR) as small as 36% achieves an average optical transmittance up to ∼90% in the visible regime, breaking the optical limits of both the unpatterned film counterpart and the thick grid counterpart (whose optical transmittance is determined by the OR). The small OR enables a low sheet resistance of ∼21.5 Ω sq^−1^. The figure of merit up to ∼17 kΩ^−1^ is superior to those of the unpatterned film counterpart, our fabricated 180 nm thick ITO, as well as most reported thick metal grid TCEs. Our ultrathin TCE, firmly attached to the substrate, is mechanically more flexible and more stable than the film counterpart and ITO. As a flexible transparent film heater, it achieves comparable or even superior heating performances with previously-reported heaters and performs well in a thermochromic test.

## Abstract 2

Patterned few nanometers thick silver films with high optical transparency, electrical conductivity, mechanical flexibility and stability.

## Graphical abstract

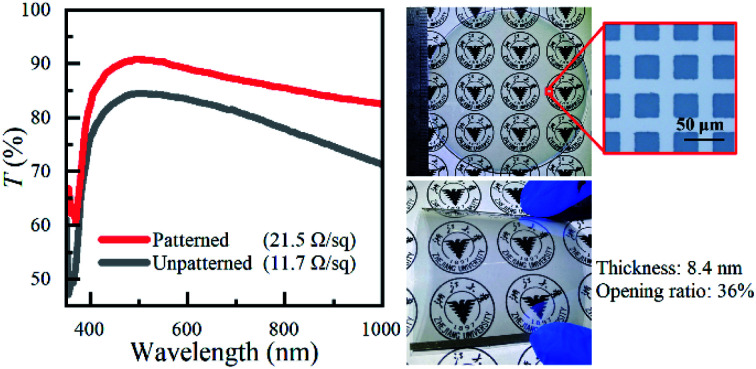

## Introduction

Transparent conductive electrodes (TCEs), which by definition transmit light (usually in the visible wavelength range) and conduct electrical current simultaneously, are essential elements for various optoelectronic devices, such as film heaters, touch panels, solar cells, and light emitting diodes (LEDs).^1^ Because of the good optical and electrical performances, indium tin oxide (ITO) has dominated the TCE field for decades.^2^ However, ITO requires high-temperature annealing after deposition and is increasingly expensive due to the scarcity of indium. Furthermore, the inherent fragility has hindered its potential applications to emerging flexible optoelectronics, which require not only high transparency and conductivity, but also strong mechanical flexibility.^3^ Indium-free transparent conductive oxides (TCOs) have relatively lower cost, but still suffer from the same problem with brittleness if relatively thick films are employed for good conductivities.^4^

In order to replace ITO and other TCOs, carbon- and metal-based alternatives have been developed.^5,6^ Carbon-based TCEs, *e.g.*, conductive polymers,^7,8^ carbon nanotubes,^9,10^ graphene,^11,12^ always have rather low carrier concentrations and thus inferior conductivities to the metallic TCEs.^1,5,6^ Bulk metals are good conductors but are opaque due to the high absorptivity and reflectivity; while micro-/nano-structured metals, such as nanowire networks,^13–15^ patterned grids,^5,6^ and ultrathin films (∼10 nm or less),^5,6,15–17^ are promising for replacing ITO because of the high light transmission and electrical conductivity, as well as the excellent mechanical flexibility. Metallic nanowire networks can be processed in solution with low cost. The nanowires are usually randomly and sparsely stacked and distributed. In this case, light can transmit through the network with low loss. However, such networks are actually discontinuous, and their conductivities are limited by wire-to-wire contact resistances. The three-dimensional (3D) geometry also enables air or chemicals to easily infiltrate into the network. Therefore, the nanowires, difficult to protect, are susceptible to chemical corrosion. We also found that the nanowire networks suffered from poor adhesion to the substrates.^18–20^ In contrast, metallic patterned grids and ultrathin films do not have those problems.^5,6,15–17^ Previously-reported metal grids had either regular patterns fabricated through electron-beam lithography or photolithography, *e.g.*, rectangles,^21–28^ modified rectangles,^29^ hexagons,^26,30^*etc.*, or irregular patterns with self-forming cracks/masks, *e.g.*, titanium oxide,^31^ egg white,^32,33^ CA200,^32^ leaf venation,^34,35^ spider's silk web,^35^ electro-spun fibers,^36^ all paint containing colloidal silicon dioxide,^37^ cosmetic containing acrylic emulsion,^37^*etc.* In general, the grid lines are made narrow enough so that light can transmit through the sufficiently large spacing without loss and meanwhile thick enough to maintain a sufficiently high conductivity.^21–27,30–37^ Because of the opaque grid lines, the optical transparency is usually limited by the opening ratio (OR), which is defined as the ratio of the space area to the unit cell area. Sheet resistances, *R*_sh_, less than 1 Ω sq^−1^ have been reported with metallic grids that have micrometer thick grid lines.^22,32,33^ In this case, the overall roughness is increased, which may cause shorting in some thin-film optoelectronics, *e.g.*, organic solar cells or LEDs. The micrometer thick grid may have serious shadow effects.^22,32,33^ In comparison, few nanometers thick metallic films in a two-dimensional (2D) morphology are much smoother. However, it is challenging to deposit smooth and continuous metal films with both high optical transmission and high electrical conductivity.^5,6,15–17^ Due to the large surface energy difference between the substrate and the metal to be deposited, the substrate usually has poor wettability to the metal, leading to 3D Volmer–Weber growth mode in the initial stage of physical vapor deposition processes.^38–41^ Fortunately, different methods have been developed to successfully suppress the 3D growth mode and evolve into a 2D morphology at an ultralow thickness, such as treating the substrate surface with seed layers or surfactants (*e.g.*, oxides,^42,43^ metals,^44,45^ molecules^46–49^), doping the metal,^50,51^ lowering the substrate temperature during deposition,^52^*etc.* It is clear that an increase in thickness results in an increase in film conductivity but a decrease in optical transmittance. However, a too thin film is also not favorable for optical transmittance because of the plasmonic absorption excited in island-like discrete metallic clusters. Only at a certain thickness, where a continuous film just develops, can the best transparency be achieved, though the transparency is still not very high absolutely because of the intrinsic metallic absorption and reflection. Hybrid TCE consisting of a 100 nm thick copper grid on top of a 2 nm thick nickel film can achieve greatly improved conductivity but degraded transmittance.^17^

Here in this work, in order to break the optical limits of both metallic patterned grids and ultrathin films mentioned above, we propose to combine their advantages and experimentally demonstrate distinct TCEs consisting of patterned few nanometers thick silver (Ag) films on both rigid and flexible substrates coated with zinc oxide (ZnO) seed layers. As far as we know, metallic grids thinner than ours have not been reported. With an OR as small as 36%, our ultrathin TCE still achieves an average optical transmittance, *T*_ave_, up to ∼90% in the visible regime, much higher than the unpatterned film counterpart (*T*_ave_ ∼ 84%) and the thick grid counterpart (whose *T*_ave_ depends on the grid OR). The small OR allows a relatively low *R*_sh_ ∼ 21.5 Ω sq^−1^. The achieved figure of merit (FoM) is up to ∼17 kΩ^−1^, which is superior to that of a 2D film counterpart without any patterns (FoM ∼ 15 kΩ^−1^) and to that of a 180 nm thick ITO (FoM ∼ 0.6 kΩ^−1^). It is comparable to and even higher than those of most thick metal grid based TCEs reported.^21,23–31^ Throughout the whole paper, the FoMs are calculated using the following widely-accepted expression:^53,54^1
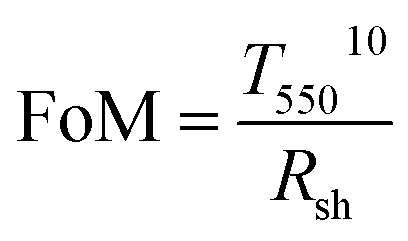
where *T*_550_ is the optical transmittance at the wavelength of 550 nm. Our patterned sub-10 nm TCE, strongly attached to the substrates, demonstrates better mechanical flexibility than the non-patterned film counterpart, let alone the brittle ITO. Finally, our TCE is explored as a flexible transparent film heater, which achieves comparable or even superior heating performances as compared with previously-reported heaters.^19,24,55–59^ Therefore, our patterned sub-10 nm Ag films are highly promising for many applications, including emerging flexible and thin-film optoelectronics (where shorting can be avoided).

## Experimental section

### Fabrication of continuous few nanometers thick Ag films

Pieces of quartz (2 cm × 2 cm), serving as rigid substrates, were ultrasonically cleaned with acetone, isopropanol, and deionized water in sequence, each for 5 min. On top of each substrate, a ∼40 nm thick ZnO film was deposited at a rate of ∼0.4 Å s^−1^ by a magnetron sputter (Kurt J Lesker PVD75; RF power: 100 W, argon pressure: 3 mTorr, room temperature). Subsequently, Ag films with different thicknesses were deposited by another sputter at 3.7 Å s^−1^ (Denton Discovery 365; DC power: 40 W, argon flow rate: 37 sccm, room temperature). With the bottom ZnO seed film, continuous ultrathin Ag films could be achieved with thicknesses of only several nanometers. For comparison, Ag films of different thicknesses were sputtered directly onto the quartz substrates without the ZnO seed layer.

### Fabrication of patterned few nanometers thick Ag films

In order to conduct patterning, UV photolithography was employed to define regular grid patterns into the photoresist (PR; AZ5214), which was spin-coated at 6000 rpm for 40 s on top of a 4′′-diameter ZnO-covered quartz substrate. After Ag film deposition according to the above sputtering recipe, the PR was ultrasonically removed by acetone, leaving a uniform grid-patterned Ag film on top of the substrate. It was very easy to conduct this lift-off process, because the PR (∼1.3 μm in thickness) was much thicker than the sub-10 nm Ag film. For further removal of the remaining solvent, the sample was ultimately cleaned with isopropanol and deionized water in sequence.

### Fabrication of flexible few nanometers thick Ag films with and without patterns and flexible transparent heaters

Pieces of PE (50 μm thick) were chosen as the substrates for flexible TCEs and transparent heaters. They were carefully attached to silicon wafers before the deposition of the ∼40 nm thick ZnO seed layer. After the patterned and unpatterned sub-10 nm Ag films were processed with the above-mentioned procedures, the PE substrates were peeled off from the wafers, resulting in flexible TCEs. Flexible transparent heaters were achieved by applying Ag conductive paste to the flexible TCEs in a two-terminal side-contact configuration, as shown in Fig. 4a.

### Characterization

Our home-built integrating sphere based spectrophotometer was employed to characterize the optical transmission spectra of our TCEs, which were normalized to those of quartz or PE substrates. The sheet resistances were measured using our home-built four-point probe system. SEM inspections were conducted with a field emission scanning electron microscope (Carl Zeiss Ultra 55). For flexibility tests, flexible TCEs were bent to different curvatures or to a fixed radius of curvature of ∼5 mm for 500 cycles *via* two conductive aluminium supports (one fixed, the other moving; the photo was shown in the inset of Fig. 3c). For each sample, the resistance between the Ag paste edge contacts was measured using a source meter (Keithley 2450). The relative changes in resistance were calculated. The temperature distributions were recorded by an infrared thermometer (Hikvision H10). For the thermochromic test, letters “ZJU” were written on the heater using a glass dip pen with thermochromic ink (AIC47634) in it. Upon powering the heater with a DC bias of 5 V, the letters were observed to fade away within 6 s.

## Results and discussion

### Few nanometers thick Ag films

Before demonstrating patterned few nanometers thick Ag films, we first investigated the development of sub-10 nm continuous Ag films without any patterns. As mentioned above, such a film with complete continuity is usually difficult to deposit due to the 3D Volmer–Weber growth mode in the initial deposition stage. In order to minimize this unfavorable stage, we introduced a ZnO seed layer between the Ag film and the quartz substrate so as to improve the wettability of the substrate. The seed layer was ∼40 nm in thickness, which was sufficiently thin and would not degrade the mechanical flexibility of the film or grid (to be shown in Fig. 3 below). Both ZnO and Ag were deposited through sputtering (Experimental section). For comparison, Ag films were also sputtered on top of bare quartz substrates.

**Fig. 1:**
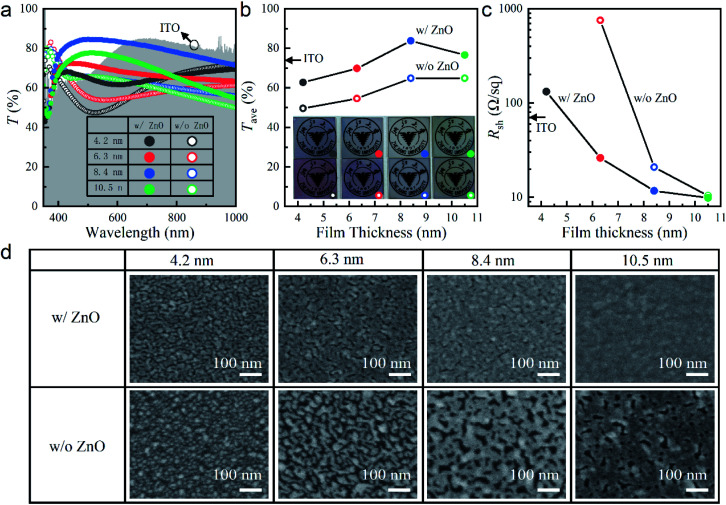
(a) Transmittance spectra, *T*(*λ*), (b) average transmittance, *T*_ave_, (c) sheet resistance, *R*_sh_, and (d) SEM images of Ag films with thicknesses of 4.2, 6.3, 8.4, and 10.5 nm deposited on quartz substrates with and without the ZnO seed layers. *T*(*λ*) (gray background), *T*_ave_ (arrow) and *R*_sh_ (arrow) of a 180 nm thick ITO film are also plotted in figures (a), (b) and (c), respectively. *R*_sh_ of the 4.2 nm Ag film on the bare quartz substrate is too large to measure.

Fig. 1a shows the measured optical transmittance spectra, *T*(*λ*), of our fabricated ultrathin Ag films with different thicknesses on top of quartz substrates with and without the ZnO seed layers. In order to quantitatively demonstrate their optical transparencies, average transmittances, *T*_ave_, were calculated with the following equation and plotted in Fig. 1b:2
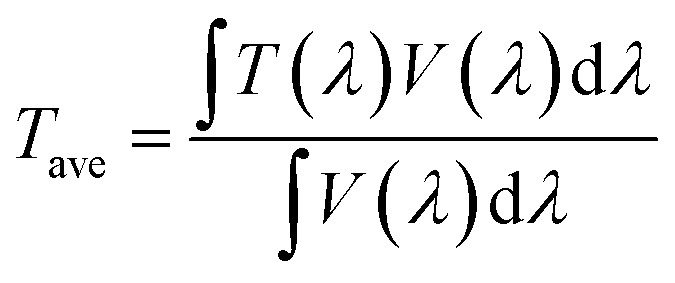
where the measured *T*(*λ*) was weighted with CIE “physiologically-relevant” luminous efficiency function, *V*(*λ*) (peaking at 555 nm and approaching zero beyond the visible wavelength range from 400 nm to 780 nm as shown in Fig. S1†). From Fig. 1a, it was seen that for an Ag film with a given thickness, the presence of the ZnO seed layer improved the optical transmission not only in the visible wavelength range from 400 nm to 780 nm but also in the invisible near-infrared range. Due to the intrinsic absorption of the ZnO seed layer, the transmittance in the ultraviolet (UV) regime was reduced, which fortunately did not affect the visual appearance. As shown in Fig. 1b, at least 12% improvement in *T*_ave_ was achieved for the seeded samples compared with the non-seeded counterparts. Therefore, the former looked more transparent than the latter, as shown in the inset of Fig. 1b. The lowest *T*_ave_ value for the 4.2 nm Ag film on the ZnO-coated quartz substrate was mainly due to the transmission dip in the visible regime. The dip indicated localized plasmonic resonances (LPRs) induced absorption, to be discussed in detail along with the morphologies of the Ag films on both bare and ZnO-seeded quartz substrates shown in Fig. 1d. The transmission dip disappeared and instead became enhanced for the 6.3 and 8.4 nm Ag films (Fig. 1a). Therefore, *T*_ave_ was improved first as the Ag thickness increased. Further increasing the film thickness degraded the visible light transmission due to the intrinsic absorption of Ag, leading to a lower *T*_ave_ value. The samples without the seed layers behaved similarly but with smaller *T*_ave_ values, as shown in Fig. 1b. This was mainly due to the much deeper transmission dips in the visible range for the 4.2 and 6.3 nm Ag films, which needed thicker absorptive Ag films to mitigate (Fig. 1a). Among all the samples, the 8.4 nm Ag thin film deposited on top of the ZnO-coated quartz substrate was the most transparent with *T*_ave_ ∼ 84%, which was ∼20% better than the film deposited on the bare substrate (Fig. 1b). Consequently, the university logo under this sample was seen the most clearly, as shown in the inset of Fig. 1b. Due to the higher transmission spectral from 400 nm to 600 nm (Fig. 1a), this sample was also superior to the 180 nm thick ITO with *T*_ave_ of only ∼74% (Fig. 1b).

In Fig. 1c, it was seen that with the ZnO seed layer, the ultrathin Ag films were more conductive than the counterparts without the seed layers. The sheet resistance, *R*_sh_, of the seeded sample dropped quickly to ∼11.7 Ω sq^−1^ when the film thickness was increased to 8.4 nm, more than six times smaller than that of the 180 nm thick ITO (*R*_sh_ ∼ 74.8 Ω sq^−1^). As the film thickness was further increased to 10.5 nm, *R*_sh_ decreased slowly to ∼10 Ω sq^−1^, to which *R*_sh_ of the sample without the seed layer approached. In this paper, the ITO used for comparison was deposited in our own laboratory through room-temperature sputtering without post-annealing (unless otherwise specified).

In order to understand the above optical and electrical performances, we investigated the effect of the ZnO seed layer on the morphological evolution of the Ag films with different thicknesses on quartz, inspected with a scanning electron microscopy (SEM) and shown in Fig. 1d. To avoid charging, part of the top surface of each sample was attached with a piece of conductive copper foil tape and electrically connected to the metallic sample holder underneath. In this case, the original unattached part of the top Ag film could be inspected clearly with the electrons. Without the Ag film, the ZnO-coated quartz substrate could not be seen clearly even with partially attached conductive copper foil tape, because of the poorer conductivity of ZnO (Fig. S2†). The insulating pure quartz was totally could not be seen. These results confirmed that Ag films was indeed deposited on the substrates and was seen from the SEM inspection. In Fig. 1d, dense Ag nanoparticles were seen on the 4.2 nm thick film on bare quartz, which became isolated nanoclusters on the 6.3 nm thick film. Upon light illumination, LPRs were excited with strong localized optical field, resulting in enhanced absorption and thus transmission dips in the visible wavelength range (Fig. 1a). The isolated geometry led to high *R*_sh_, which was too large to measure at the minimal thickness of 4.2 nm (Fig. 1c). At the thicknesses of 8.4 and 10.5 nm, the nanoclusters coalesced into sparse and dense networks, respectively. Consequently, the LPR-induced transmission dips disappeared with improved transmittance (Fig. 1a), while with increased electron transport paths, *R*_sh_ was greatly reduced (Fig. 1c). In great contrast, ZnO had a much better wettability for Ag deposition. The initial Ag atoms were immobilized on the ZnO surface for adsorbing more atoms. For the Ag film as thin as 4.2 nm, large nanoclusters instead of small nanoparticles appeared. Hence, *R*_sh_ was measurable (Fig. 1c). The optical transmittance was significantly improved compared with the same Ag film deposited on the bare substrate (Fig. 1a). As the thickness increased to 8.4 nm, the Ag nanoclusters quickly developed into a 2D nanosheet. Complete coalescence occurred when the thickness was further increased to 10.5 nm. The close values of *R*_sh_ for these two samples in Fig. 1c illustrated that the 8.4 nm film was thick enough to form a continuous layer. It was also sufficiently thin to minimize the intrinsic absorption of Ag and achieve the higher *T*_ave_ than the 10.5 nm film (Fig. 1b).

### Patterned few nanometers thick Ag films

**Fig. 2:**
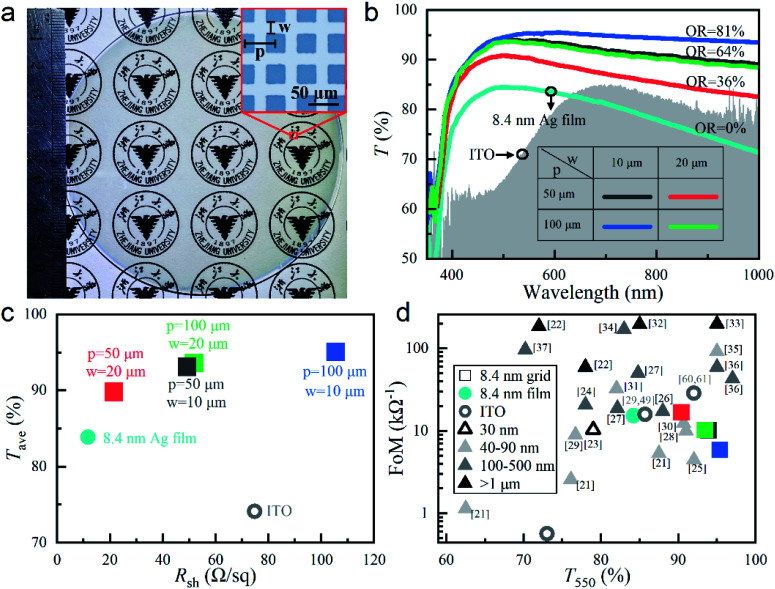
(a) Optical photograph of our fabricated 8.4 nm thick Ag grid TCE on a 4′′ ZnO-coated quartz substrate; the microscopic image in the inset shows the grid period of *p* = 50 μm and the Ag line width of *w* = 20 μm, which define the opening ratio of OR = 36%. (b) Transmittance spectra, *T*(*λ*), and (c) average transmittance, *T*_ave_, *versus* sheet resistance, *R*_sh_, for the 8.4 nm thick Ag grid TCEs with different sets of *p* and *w* (*i.e.*, different ORs) on top of the ZnO-coated quartz substrates, as well as our deposited 180 nm thick ITO. (d) FoMs *versus* the transmittance at 550 nm, *T*_550_, of our 8.4 nm thick Ag film and grid TCEs, our deposited 180 nm thick ITO (unlabeled), previously-reported ITO^29,49,60,61^ and those of some metallic meshes with different thicknesses reported previously (indicated with different triangle symbols).^21–37^

In order to further improve the transparency of the 8.4 nm thick Ag film on top of the ZnO seed layer, periodically arranged blank squares with 100% transmission were introduced into the top Ag film, which were predefined in a photomask for photolithography. Since all the fabrication processes (described in the Experimental section in detail) were scalable, such ultrathin Ag grid TCEs could be made as large as possible. Limited by our laboratory facilities, we only demonstrated in Fig. 2a an 8.4 nm thick Ag grid TCE on a ZnO-coated quartz substrate with the diameter as large as 4′′. Its opening ratio, OR, was 36% with grid period *p* = 50 μm and line width *w* = 20 μm (inset of Fig. 2a). With patterning, the university logos under this grid sample looked much clearer than that under the film sample with the same Ag thickness (inset of Fig. 1b). This was well verified by the improved transmittance spectrum over the whole considered wavelength range even for the sample with OR as low as 36%, as demonstrated in Fig. 2b. When *p* became larger and/or *w* became smaller, OR would increase. In this case, more light could transmit through the enlarged spacing, where the ZnO coating was exposed. Because of the much better transmission of the pure ZnO coating in the grid spacing than the combined Ag and ZnO double-layer in the grid line (Fig. S3a†), the whole transmittance spectra became enhanced, especially in the near-infrared wavelength range. As shown in Fig. S3a,† the transmittance spectra of our grid samples, *T*(*λ*), could be well predicted with their ORs and the transmittance spectra of both the grid spacing, *T*_space_(*λ*) and the grid line (*i.e.*, the 8.4 nm Ag film on the ZnO seed layer), *T*_line_(*λ*), through the following equation:

3 *T*(*λ*) = OR × *T*_space_(*λ*) + (1 − OR) × *T*_line_(*λ*)

For previously-reported thick metallic grids, the transmittances were always limited by their ORs,^21,30^ while our ultrathin Ag grids achieved much higher transmittance than their ORs (though the exposed ZnO coating might introduce some reflection and absorption). This totally benefited from the contribution of the highly transparent 8.4 nm thick Ag film on the ZnO seed layer. According to eqn (3), *T*(*λ*) could be further increased if the ZnO seed layer was simultaneously patterned into grid with the top Ag film (where *T*_space_(*λ*) = 1). However, it could be predicted that *T*(*λ*) would not improve too much, especially in the visible and near-infrared wavelength range, because *T*_space_(*λ*) was already close to 1 in this wavelength range for the ∼40 nm thick ZnO layer (Fig. S3b†). The sample with the smallest OR = 36% would have the minimal influence of the spacing transmission on its overall grid transmittance. That is why we did not conduct patterning on the ZnO seed layer.

As shown in Fig. 2c, *T*_ave_ calculated with eqn (2) increased with the increasing OR due to the improving transmittance spectra (Fig. 2b). On the other hand, *R*_sh_ also increased, because there were less electron transport paths in a unit area for the sample with a larger OR. From Fig. 2b and c, it was seen that the samples with the same OR = 64% (*p* = 50 μm, *w* = 10 μm; *p* = 100 μm, *w* = 20 μm) achieved almost the same *T*(*λ*), *T*_ave_ and *R*_sh_, which could indicate the dependence of the OR value rather than the specific grid geometry. For the sample with OR = 36%, *R*_sh_ ∼ 21.5 Ω sq^−1^ was not degraded too much from that of the Ag film and was still much smaller than that of the 180 nm thick ITO.

In order to highlight the optical performances, FoMs *versus T*_550_ (instead of *R*_sh_) were plotted in Fig. 2d, where FoMs of various reported grid TCEs with different thicknesses were included.^21–37^ Among our fabricated 8.4 nm thick Ag grid and film TCEs, the one with OR = 36% achieved the highest FoM of ∼17 kΩ^−1^, because of the best balance between *R*_sh_ and *T*_550_. Such a FoM could not compete with those achieved by >1 μm ultrathick grid TCEs with ultralow *R*_sh_'s,^22,32,33^ but was comparable to and even higher than those obtained by most grid TCEs with intermediate thicknesses between 30 nm and 500 nm.^21,23–31^Fig. 2d also shows that our ultrathin grid TCE had a very high transmittance of *T*_550_ ∼ 90%, which only a few thick grid TCEs could surpass.^25,28,30,33,35,36^ The thinnest TCE we could find in the literature was 30 nm,^23^ which had an FoM of ∼10 kΩ^−1^ and a *T*_550_ of less than 80%. In comparison, our fabricated 8.4 nm thick Ag grid TCE with OR = 36% had much better performances in both FoM and *T*_550_. Considering the poor performance of our deposited 180 nm thick ITO, some better representative results were also included in Fig. 2d for further comparison, *i.e.*, *T*_550_ = 85.7%, FoM = 15.9 kΩ^−1^,^29,49^ and *T*_550_ = 92%, FoM = 28.9 kΩ^−1^.^60,61^ The FoM of the 8.4 nm thick Ag grid TCE with OR = 36% was much higher than that of our deposited ITO (FoM ∼ 0.6 kΩ^−1^) but was between the previously-reported ITO data in terms of both *T*_550_ and *R*_sh_. This indicated that our ultrathin Ag grid TCEs was very competitive to replace ITO.

Furthermore, our 8.4 nm thick Ag film and grid were firmly attached to the ZnO-seeded substrates, which could not be peeled off with scotch tape (Video S1, ESI†). This adhesion was much stronger than that of the nanowire networks, which could be easily removed from the substrates according to our previous experience.^18–20^ The ultrathin geometry could be protected from corrosion simply with an additional dielectric coating, *e.g.*, a 10 nm thick ZnO film, while such a simple protecting method was not applicable to the randomly distributed nanowire networks. The few nanometers thick film or grid was also more advantageous in many applications, *e.g.*, thin-film solar cells and LEDs, where shorting might be avoided.

### Flexible patterned few nanometers thick Ag films

**Fig. 3:**
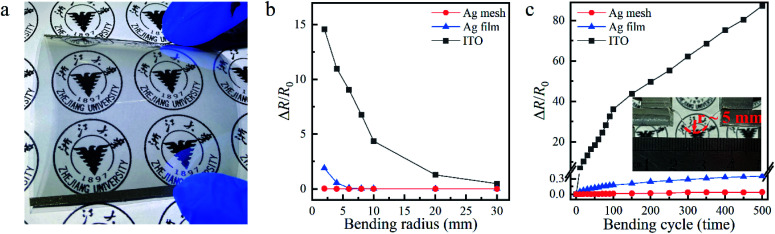
(a) Optical image of a flexible patterned 8.4 nm Ag film fabricated on a ZnO-coated PE substrate (6 cm × 6 cm). Relative change in resistance, Δ*R*/*R*_0_, as functions of (b) bending radius and (c) bending cycles at the bending radius of ∼5 mm for our 8.4 nm Ag grid TCE (red dots), film TCE (blue triangles), as well as the 180 nm thick ITO (black squares).

Our few nanometers thick Ag films with or without grid patterns could be easily prepared on any substrates. In this work, we fabricated them on flexible polyethylene (PE) substrates with the same procedure for the samples on rigid quartz substrates. Fig. 3a shows a flexible patterned 8.4 nm thick Ag film with OR = 36% fabricated on a ZnO-coated PE substrate as large as 6 cm × 6 cm. It was as conductive and transparent as the rigid one shown in Fig. 2a. To investigate its mechanical performance, flexibility tests were conducted with the setup shown in the inset of Fig. 3c. For comparison, the mechanical flexibilities of the unpatterned 8.4 nm thick Ag film and the 180 nm thick ITO were also tested with the same setup. The experimental details were described in the Experimental section. Relative changes in resistance of the three samples after bending were recorded as Δ*R*/*R*_0_ = (*R* − *R*_0_)/*R*_0_ (where *R*_0_ and *R* were measured resistances of the sample before and after bending) and plotted in Fig. 3b and c as functions of bending radius and cycles, respectively. As shown in Fig. 3b, the resistance of our 8.4 nm thick Ag grid TCE kept almost unvaried even when the bending radius was reduced to as small as ∼2 mm. Without patterning, the resistance of the 8.4 nm thick Ag film increased when the bending radius decreased from ∼6 mm to ∼2 mm. This meant that the grid pattern could well relax the stress accumulated during small-radius bending. As expected, the resistance of the brittle ITO increased dramatically as the bending radius decreased, due to the formation and propagation of microscopic cracks. On the other hand, as shown in Fig. 3c, our 8.4 nm thick Ag grid TCE was able to withstand 500 cycles of bending at a curvature radius of ∼5 mm. Its resistance was almost unchanged. However, for the unpatterned Ag film TCE and ITO, the bending stress could not be relaxed, and the resistances rose slowly and quickly, respectively, as the bending cycle increased. All these results showed that the ∼40 nm thick ZnO seed layer did not affect the mechanical performances of our TCEs. Due to the excellent mechanical flexibility and stability, our patterned sub-10 nm Ag film on the ZnO seed layer could potentially be applied to the emerging hot area of flexible electronics.

### Flexible transparent heaters based on patterned few nanometers thick Ag films

**Fig. 4:**
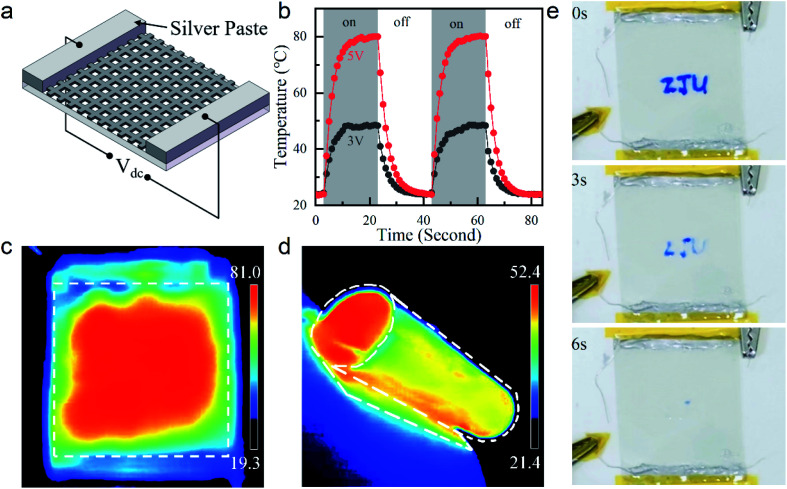
Flexible thin-film transparent heaters based on our patterned 8.4 nm thick Ag film TCEs with OR = 36% on ZnO-coated PE substrates: (a) schematic illustration; (b) time-dependent central temperatures under different DC biases of *V*_dc_ = 3 V (black) and 5 V (red); infrared images of (c) a unbent heater (2 cm × 2 cm, *V*_dc_ = 5 V) showing a uniform temperature distribution, and (d) a heater bent into a roll (6 cm × 6 cm, *V*_dc_ = 5 V); (e) thermochromic test of our flexible transparent heater (2 cm × 2 cm, *V*_dc_ = 5 V), on which “ZJU” written by the thermochromic ink faded away within 6 s after the DC voltage was applied.

To explore a practical application, we employed our few nanometers thick Ag grid TCE as a flexible transparent thin-film heater. As schematically shown in Fig. 4a, a constant direct current (DC) voltage (*V*_dc_) was applied to the Ag paste contacts on the opposite edges of the grid TCE, where heat was generated through the Joule effect. Fig. 4b shows time-dependent temperature responses of our 8.4 nm thick Ag grid TCE with OR = 36% and area of 2 cm × 2 cm at *V*_dc_ = 3 and 5 V. The temperature at the sample center was recorded. A temperature up to ∼80 °C was achieved when the heater was powered by a 5 V bias. It was comparable and even superior to those obtained by many other previously-reported transparent thin-film heaters (based on *e.g.*, metal nanowires, doped ZnO film, carbon nanotubes, *etc.*) with similar sizes and under the same biases.^19,24,55–59^ Our device showed rapid responses upon being powered and unpowered. Regardless of the applied voltages, it took ∼6 s for the temperature of our heater to rise from 10% to 90% of its maximum when the bias was turned on; and took another ∼7 s to drop from 90% to 10% when the bias was turned off. The homogeneous steady-state temperature profile shown in Fig. 4c illustrated excellent thermal and electrical conductivities of our device with uniform grid patterns. Our transparent heater also worked well in a curling state, as demonstrated in Fig. 4d. Here, a larger heater was fabricated based on a 6 cm × 6 cm ultrathin Ag grid TCE for the convenience of being made into a roll. With the same biases, it could not reach the steady-state temperature as high as the small one due to the reduced power density and increased heat exchange with the relatively cold surroundings. The rolled heater also exhibited a uniform heat distribution, indicating its excellent mechanical flexibility. Finally, a thermochromic test was conducted to show the effectiveness of our transparent thin-film heater based on the 8.4 nm thick Ag grid TCE. As shown in Fig. 4e, before heating, letters “ZJU” were written on the heater using a thermochromic ink. Upon biased with *V*_dc_ = 5 V, heat was generated and the letters faded away immediately. Within 6 s, the letters completely disappeared.

## Conclusions

In conclusion, we have demonstrated experimentally distinct TCEs based on patterned few nanometers thick Ag films on ZnO-seeded rigid and flexible substrates. The grid lines are completely continuous, but only 8.4 nm thick. As far as we know, even thinner metallic grids have not been reported. Owing to the high transparency of both the ultrathin grid lines and the grid spacing, our TCE with an OR as small as 36% achieves a *T*_ave_ up to ∼90% in the visible regime, much higher than the unpatterned film counterpart with *T*_ave_ ∼ 84% and the thick grid counterpart with *T*_ave_ mainly limited by the OR. The small OR or wide grid lines enable a relatively low *R*_sh_ ∼ 21.5 Ω sq^−1^. The achieved FoM is up to ∼17 kΩ^−1^, which is superior to that of a 2D film counterpart without any patterns (FoM ∼ 15 kΩ^−1^) and to that of our fabricated 180 nm thick ITO (FoM ∼ 0.6 kΩ^−1^). It is comparable to and even higher than those of most reported thick metal grid based TCEs.^21,23–31^ Our patterned sub-10 nm TCE keeps almost unvaried in terms of conductivity when bent with a curvature radius as small as ∼2 mm or after being bent 500 times at a bending radius of ∼5 mm. Such mechanical flexibility and stability are better than the non-patterned film counterpart, let alone the brittle ITO. Our TCE can be employed as a high-performance flexible transparent film heater, which achieves comparable or even superior heating performances with previously-reported heaters,^19,24,55–59^ and performs well in a thermochromic test. Furthermore, it has much stronger attachment to the substrate than the nanowire networks according to our previous experience.^18–20^ Because of the ultrathin geometry, our TCE can be easily protected from corrosion and potentially be incorporated into emerging flexible and thin-film optoelectronics.

## Conflicts of interest

There are no conflicts to declare.

## Supplementary Material

RA-011-D1RA00549A-s001

RA-011-D1RA00549A-s002
