# Supplementary material for: Patterned few nanometer-thick silver films with high optical transparency and high electrical conductivity
Source: RSC Adv. 2021 Mar 19;11(19):11481–9. doi: 10.1039/d1ra00549a (PMC8695985; doi:10.1039/d1ra00549a)
Supplement: RA-011-D1RA00549A-s001 [file RA-011-D1RA00549A-s001.pdf]

Electronic Supplementary Material (ESI) for RSC Advances.
This journal is © The Royal Society of Chemistry 2021

Supporting Information

Patterned Few Nanometers Thick Silver Films with High Optical Transparency and High Electrical Conductivity

Xie He,[a] Qijie Cao,[a] Jing Pan,[a] Liu Yang*[a,b] and Sailing He*[a,b,c]

[a] Centre for Optical and Electromagnetic Research, National Engineering Research Center for Optical Instruments, Zhejiang University, Hangzhou 310058, China. E-mails: optyang@zju.edu.cn; sailing@zju.edu.cn

[b] Ningbo Research Institute, Zhejiang University, Ningbo 315100, China.

[c] JORCEP, School of Electrical Engineering, Royal Institute of Technology (KTH), S-100 44 Stockholm, Sweden.

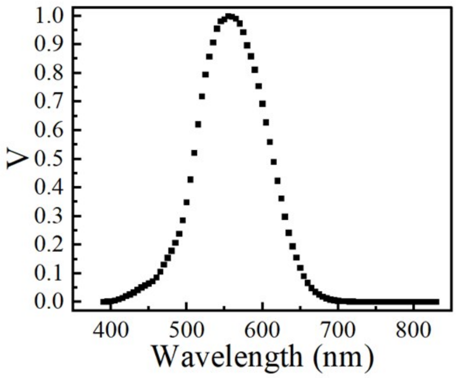

Fig. S1. CIE "physiologically-relevant" luminous efficiency function (2 degree). Data source: http://www.cvrl.org/.

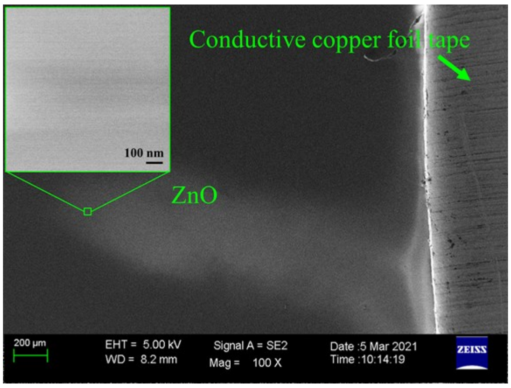

Fig. S2. SEM image of the quartz substrate coated with a ~ 40-nm thick ZnO seed layer. The ZnO could not be seen clearly because of its poor conductivity. The inset is a zoomed-in image of ZnO..

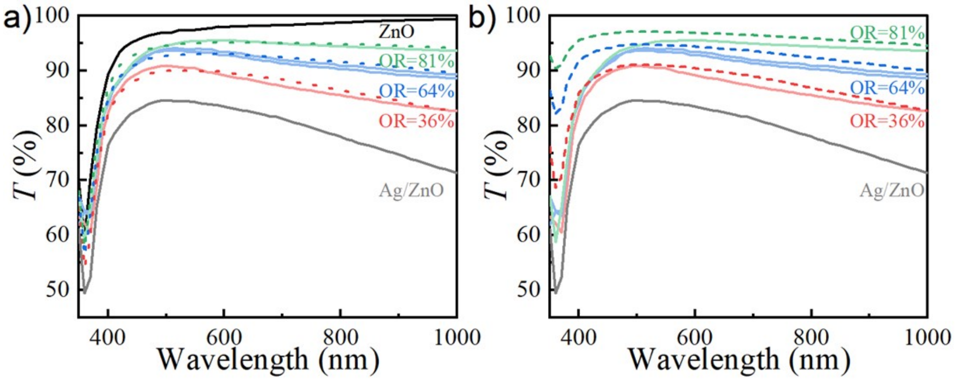

Fig. S3. Comparison of measured (solid curves) and calculated (dash curves) transmittance spectra of our patterned sub-10 nm ultrathin Ag films with OR = 36% (p = 50 µm, w = 20 µm), 64% (p = 50 µm, w = 10 µm; p = 100 µm, w = 20 µm), and 81% (p = 100 µm, w = 10 µm): a) measured transmittance spectra of the grid spacing (i.e., ~ 40-nm thick ZnO seed layer; black curve) and the grid line (i.e., the 8.4-nm ultrathin Ag film on top of the ZnO seed layer; Ag/ZnO; grey curve) are employed in the calculation; b) the grid spacing transmittance is assumed to be 1 in the whole wavelength range in the calculation. Equation (2) in the main text are employed for the calculations.
